# Laboratory Surveillance of Dengue in Argentina, 1995–2001

**DOI:** 10.3201/eid0906.020483

**Published:** 2003-06

**Authors:** Gabriela Avilés, Maria Valeria Paz, Griselda Rangeon, Marie Y. Ranaivoarisoa, Nora Verzeri, Sandra Roginski, Pablo Baroni, Delia Enria

**Affiliations:** * Instituto Nacional de Enfermedades Virales Humanas (INEVH), Pergamino, Argentina; † Ministerio de Salud, Salta, Argentina; ‡ Ministerio de Salud, Formosa, Argentina; § Ministerio de Salud, Buenos Aires, Argentina; ¶ Ministerio de Salud, Misiones, Argentina

**Keywords:** Dengue, laboratory surveillance, Argentina, dispatch

## Abstract

Local transmission of dengue fever virus in Argentina is increased by the presence of *Aedes aegypti* mosquitoes and dengue outbreaks in neighboring countries. From 1995 to 2001, a laboratory-based active surveillance program detected 922 dengue cases. Indigenous transmission involving dengue-1 and -2 serotypes was confirmed only in subtropical areas in northern Argentina.

The mosquito *Aedes aegypti*, which carries dengue fever virus (DENV), reinfested Argentina in 1986 and is well established in the subtropical and temperate areas of the country (*1*); DENV infection was first diagnosed in the laboratory in Argentina in 1997 (*2*). Early evidence of sporadic DENV-2 circulation was detected in northwestern Argentina in 1997 in a subtropical area; 19 of 404 indigenous cases were confirmed (*2*). Dengue surveillance in Argentina was organized as a collaboration of the National Department of Epidemiology, National Ministry of Health, provincial departments of epidemiology, national and provincial programs of vector control, and DENV laboratory network. (The DENV laboratory network is coordinated by the Instituto Nacional de Enfermedades Virales Humanas “Dr. Julio I. Maiztegui” [INEVH], the national reference center of DENV diagnosis in Argentina, and the Pan American Health Organization/World Health Organization [PAHO/WHO] Collaborating Center in Viral Hemorrhagic Fevers and Arboviruses.) This network was established in the area at high risk for DENV in Argentina based on the distribution of *Aedes aegypti* from the north to 35°S, the latitude of Buenos Aires (*1*). Surveillance of imported cases has also been performed in areas with no *Ae. aegypti* infestation because of travelers from areas with DENV transmission who reported DENV-like symptoms. Laboratory-based surveillance has been closely coordinated between epidemiologic and clinical surveillance and vector control measures. Goals of the laboratory network include providing early information on timing and location of transmission, disease severity, and serotypes and genotypes present; predicting transmission; and guiding implementation of clinical and vector control measures. Actions to alleviate DENV are tailored to each region, with the size of the country, variable geographic characteristics, funding, and size of the population at risk taken into account. These actions include vector control, health education, community participation, adequate garbage handling, and adequate water supply. Although the strategy is coordinated at a national level, many of the actions are decentralized to the provincial and municipal levels.

This system enabled us to detect an outbreak in the same subtropical area in northwestern Argentina several months after the first DENV introduction in 1997. Some ongoing, undetected, transmissions may have occurred because the same DENV serotype was circulating. However, clinical surveillance did not detect cases compatible with DENV during those months, and laboratory results were negative. We believe that this outbreak could represent new activity because continuous transmission was suspected (although not confirmed) in neighboring countries. This area of Argentina has a continuous movement of persons across the borders, and imported cases were diagnosed before the outbreak.

DENV-2 was isolated for the first time in the country during an outbreak that affected only the Salta Province in 1998 (*3*). All cases identified then were classified as dengue fever by using PAHO/WHO criteria (*4*). DENV cases caused by DENV-2 had also been diagnosed in Bolivia in 1996 and 1997 (*5*). In 1999 and 2000, an outbreak of at least 27,000 cases of DENV-1 occurred in Paraguay. Evidence suggests that >100,000 cases of DENV-1 may have occurred in the Asuncion District, Paraguay (*6*). This outbreak spilled over into Argentina, where several cases occurred in the northern part of the country. We summarize the results of 6 years of surveillance and, for the first time, document the circulation of two DENV serotypes in Argentina.

## The Study

We defined a notified case as any patient whose illness was considered compatible with DENV by a health professional; samples from these cases were sent to provincial laboratories or directly to the national reference laboratory. Case definitions used for probable and confirmed cases of dengue fever, dengue hemorrhagic fever, and dengue shock syndrome were those proposed by PAHO (*4*). Because DENV transmission was not endemic in Argentina, laboratories checked continuously for the further introduction of the virus. Because the introduction of DENV was relatively recent, the total population infected relatively small, and no continuous transmission was detected, all positive serologic samples obtained at the provincial levels were sent to INEVH to be confirmed by other techniques. If a bigger, confirmed outbreak occurs in the future, we anticipate that a small number of positive samples would be evaluated at INEVH. During interepidemic periods, we conducted laboratory surveillance of febrile syndromes of undetermined etiology. Samples obtained from our surveillance program for diseases that are clinically similar to DENV (e.g., rubella, measles, Argentine hemorrhagic fever, leptospirosis and other illnesses fulfilling criteria for viral hemorrhagic fevers) were also tested for DENV.

Local-level physicians in Argentina were familiarized with the case definitions for DENV syndromes from published guidelines (*4*). After the discovery of the first occurrence of the virus and the organization of the national DENV surveillance system, the training of local healthcare personnel, including physicians and epidemiologists, was intensified through periodic courses, meetings, and seminars. In small localities, such as those in the north of the country, only a few hospitals exist in each locality, and most of them are government-owned; therefore, all personnel were informed and were willing to participate. In bigger provinces, such as Buenos Aires, the healthcare system is organized into regions, each of which has several public hospitals, but all of these hospitals are supported by a provincial department of epidemiology. Periodic meetings held to inform and train staff were quite efficient. We found no evidence of any physicians unwilling to participate in the system.

The laboratory method used in Argentina has been described in previous reports (*2*,*3*,*7*,*8*). Briefly, serum samples taken from patients with suspected DENV infection 5 days after onset of symptoms (late acute) were tested weekly during the summer and fall and monthly during the rest of the year at provincial laboratories. The laboratories used a commercial kit (ultramicro enzyme-linked immunosorbent assay capture immunoglobulin [Ig] M dengue test, Instituto Pedro Kouri, Havana, Cuba) or IgM antibody capture enzyme-linked immunosorbent assay (MAC-ELISA). All positive samples and at least 10% of negative samples were sent to INEVH for quality control and confirmation. When the surveillance system was first initiated, all negative samples were tested at the national reference center. At INEVH, samples were tested by MAC-ELISA, plaque reduction neutralization test (PRNT), hemagglutination inhibition (HI) test, viral isolation in C6/36 cells, and immunofluorescence detection (using monoclonal antibodies obtained from PAHO), or by polymerase chain reaction (PCR) techniques, according to the date of onset. In addition, the capsid/premembrane and envelope/nonstructural protein 1 regions of viral isolates were sequenced to determine the genotypes. Acute-phase serum samples (<5 days after onset of symptoms) were sent directly to INEVH and assayed for viral isolation or PCR; samples >5 days of onset were tested by MAC-ELISA for IgM antibodies. During the convalescent-phase period, a second sample was obtained from patients with positive results for DENV by MAC-ELISA and from most patients with acute-phase samples (with results either positive or negative by viral isolation or PCR); these samples were tested along with the acute-phase samples by using PRNT or HI tests. Serial dilutions of each patient’s serum were tested against the four DENV serotypes for 80% identity by PRNT or HI tests. The DENV-1 HAW, DENV-2 NGC, DENV-3 H87, and DENV-4 H241 strains were obtained from the Dengue Branch, Centers for Disease Control and Prevention, San Juan, Puerto Rico. Antigens from mice brains infected with each DENV were prepared at INEVH by using the sucrose-acetone method and used in MAC-ELISA and HI tests. According to the IgG titers by PRNT or HI tests, DENV cases were classified as primary or secondary. Primary cases were indicated by titers <160 in the acute-phase sample (<5 days after onset of symptoms), titers <1,280 in the late acute- or convalescent-phase sample, and low or null cross-reactivity among the different DENV serotypes. Secondary cases were indicated by titers >160 in the acute-phase sample, titers >2,560 in the late acute- or convalescent-phase sample, and high cross-reactivity among the different DENV serotypes.

**Figure 1:**
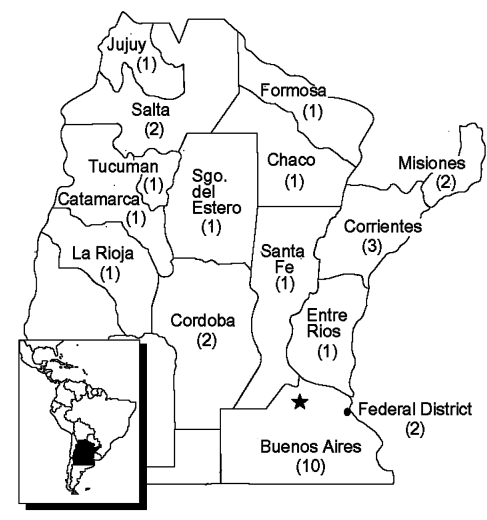
Dengue laboratory network, Argentina.

A laboratory network including all provinces at risk for DENV (according to *Ae. aegypti* distribution) was established in 1998 (Figure 1). The national reference laboratory, which is self-sufficient for production of key reagents (such as antigens and antiserum), participates in the proficiency tests organized under PAHO/WHO and maintains country-proficiency tests on a continuing basis. Commercial kits were evaluated at the national reference center before being used in national programs. Surveillance for yellow fever, St. Louis encephalitis, West Nile virus, and other flaviviruses were also incorporated into DENV diagnostic protocols.

Thirty laboratories were designated by the National Ministry of Health, the provincial ministries of health, and the local municipalities to integrate the laboratory network (Figure 1). Staff persons from 15 of those regional laboratories were trained on DENV diagnosis at INEVH, and 12 were actively working on DENV serologic surveillance. Courses and rotations were part of the training ongoing since the network of laboratories was organized. These 12 laboratories were evaluated on IgM detection by INEVH and demonstrated good concordance. The remaining laboratories had difficulties in obtaining reagents, keeping staff because of lack of funding, and other operational problems, so they could not sustain the work through the entire surveillance period. Training was emphasized, and INEVH focused on the problems of these laboratories to maintain high quality control. Staff from all 30 laboratories attended annual meetings at which the results and problems concerning the organization of the laboratory, clinical, and epidemiologic DENV surveillance were discussed. Laboratories sent the samples directly to INEVH when they were unable to carry out sample testing on their own.

During the epidemiologic surveillance of cases compatible with DENV from December 1995 to December 2001, our laboratory received 493 serum samples from travelers returning to Argentina with suspected DENV (Table 1) and from case-patients with no epidemiologic data. Cases were classified as imported or indigenous as a result of epidemiologic analysis, considering travel histories in the 3 weeks before onset of illness. Of 226 positive case-patients, 150 reported travel histories to Paraguay (127 cases), Brazil (11 cases), Honduras (3 cases), Venezuela (3 cases), Ecuador (1 case), Mexico (2 cases), Dominican Republic (1 case), Puerto Rico (1 case), and the Virgin Islands (1 case). Seven other cases were “imported” to other provinces inside the country during the DENV outbreaks of 1998 and 2000. No epidemiologic data were available for the remaining 69 cases. During the DENV outbreak in Salta Province in 1998 (*4*), 21 persons reported that they had traveled to Bolivia. We could not determine whether infection occurred in Bolivia or Argentina, so these cases were considered as probably imported but were included in the total number of cases (378) for the outbreak (Table 2). Imported cases were detected in different provinces throughout the country in different years: Salta (1996, 2000), Buenos Aires (1997–2000), Santa Fe (1997, 1999, 2000), Misiones (1998, 2000), Jujuy (1998, 2000), Cordoba (1998, 2000), Rio Negro (1998), Tucuman (1999), Chaco (2000), Corrientes (2000), and Formosa (2000); these cases involved either DENV-1, DENV-2, or DENV-3 serotypes (Table 1).

**Table 1:** Imported dengue virus cases and cases with no epidemiologic data, Argentina, 1995–2001^a^

Year	MAC-ELISA^b^	PRNT^b^	HI^b^	Viral isolates + amplicons^b^	DENV serotype^c^	Total^b^ by all techniques
1995	0/18	ND	ND	ND	–	0/18
1996	0/28	ND	ND	1/1	DENV-3	1/28
1997	4/16	2/4	ND	ND	–	4/16
1998	22/64	14/17	ND	ND	DENV-1,2	27/68
1999	17/37	12/12	ND	0/2	DENV-1	23/45
2000	142/307	9/10	43/44	19/31	DENV-1	162/279
2001	9/9	3/3	2/2	ND	–	9/39
Total	194/479	40/46	45/46	20/34	–	226/493^d^

^a^DENV, dengue fever virus; MAC-ELISA, immunoglobulin M antibody capture enzyme-linked immunosorbent assay; PRNT, plaque reduction neutralization test; HI, hemagglutination inhibition test; ND, not done. ^b^Positive/tested. ^c^In most cases, the serotype was determined only by serologic testing unless we indicate otherwise that virus isolation or polymerase chain reaction was also used. In many of these cases, the serotype could not be determined because of cross-reactions in serologic tests. ^d^Some samples were tested by one technique, and others were tested by two or more techniques; therefore, these numbers do not represent the total obtained by adding the rows.

**Table 2:** Indigenous dengue virus cases in Argentina, 1997–2001^a^

Year	Province	MAC-ELISA^b^	PRNT^b^	HI^b^	Viral isolates + amplicons^b^	DENV serotype ^b^	Total^b^ by all techniques
1997	Salta	19/387	18/19	ND	1/36	DENV-2	19/404
1998	Salta	359/589	139/143	79/80	5/112	DENV-2	378/701
	Several	0/438	0/56	ND	ND	—	0/457
1999	Salta	5/55	1/1	ND	0/15	—	5/55
	Several	0/171	0/14	ND	0/4	—	0/173
2000	Jujuy	2/63	ND	2/2	2/7	DENV-1	6/72
	Formosa	37/89	1/31	8/21	7/57	DENV-1	50/195
	Misiones	229/442	ND	25/27	9/25	DENV-1	238/469
	Several	0/223	ND	0/19	0/20	—	0/249
2001	Several	0/176	ND	0/2	0/46	—	0/212
Total		651/2,633	159/264	114/151	24/322		696/2,987^c^

^a^DENV, dengue fever virus; MAC-ELISA, immunoglobulin M antibody capture enzyme-linked immunosorbent assay; PRNT, plaque reduction neutralization test; HI, hemagglutination inhibition test; ND, not done. ^b^Positive/tested. ^c^Some samples were tested by only one technique, others were tested by two or more techniques; thus, these numbers do not represent the total obtained by adding the rows.

**Figure 2:**
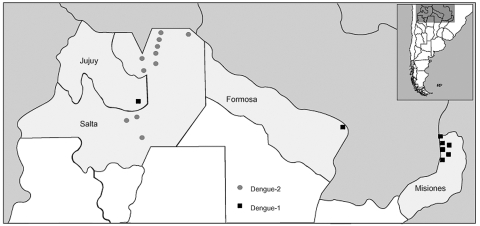
Geographic distribution of dengue cases in Argentina, 1997–2001.

From January 1997 to December 2001, a total of 2,987 serum samples were tested for suspected DENV from provinces in the subtropical (north) and the temperate (central) areas of the country; 696 samples were positive (Table 2, Figure 2). Of these cases, 378 occurred during an outbreak in Salta Province that occurred from January 3 to May 31, 1998, and was caused by DENV-2 (*3*,*7*) (Table 2, Figure 2). During the Salta outbreak, men and women were equally affected. Prevalence in adults 15–79 years of age (82.5%), was higher compared to that in children <14 years of age (17.5%). Because most samples came from adults, the disease may have been subclinical or undetected in most children. The most affected locality in this region was Tartagal (22°S, 63°W), which had an incidence rate of 67.5 cases per 10,000 inhabitants. Serologic testing by PRNT or HI for all four DENV serotypes in 154 cases from this outbreak resulted in 38 (24.7%) that showed primary responses and 84 (54.5%) that showed secondary responses. The secondary cases were considered to be indigenous because none of these patients had traveled in the previous 3 weeks. We did not gather any information about traveling before those 3 weeks, so whether the patients were infected by another flavivirus in Argentina or in any other country before that is unknown. The remaining 32 cases (20.7%) with borderline IgG titers could not be classified. Previous exposure to other flaviviruses (e.g., St. Louis encephalitis or yellow fever likely due to vaccination) explained 83% of the secondary serologic patterns; the rest remained unexplained, suggesting the unrecognized occurrence of previous infection with other DENV serotypes or flaviviruses other than St. Louis encephalitis or yellow fever (*7*). The second more important cluster of DENV cases occurred during a DENV-1 outbreak in 2000 that affected Misiones, Formosa, and Jujuy Provinces (Table 2, Figure 2), causing 294 cases from February 15 to April 22. Most affected localities were Clorinda (Formosa Province, 25°S, 57°W), with an incidence of 12.3 cases per 10,000 inhabitants and Iguazu (Misiones Province, 25°S, 54°W), with an incidence of 51.7 cases per 10,000 inhabitants.

DENV isolated in Argentina were also characterized molecularly. DENV-2 isolated in Salta in 1997 and 1998 showed 98% to 99% nucleotide identity with DENV-2 isolated from Brazil in 1990 and Jamaica in 1983 (Avilés et al., unpub. data). DENV-1 isolated in 2000 from Misiones, Formosa, and Jujuy Provinces showed 95% identity with a DENV-1 strain isolated from French Guiana in 1989 and 91% identity with a DENV-1 strain isolated from the Caribbean (Jamaica) in 1977 (*8*).

## Conclusions

Laboratory surveillance in Argentina enabled detection of an early circulation of DENV in 1997 and confirmation of two DENV outbreaks that occurred from 1998 to 2000. This surveillance system identified 922 laboratory-diagnosed cases of DENV (696 indigenous, 157 imported, and 69 unknown) during the period of study. We obtained 38 DENV-1, 6 DENV-2, and 1 DENV-3 isolates or PCR amplicons. Imported cases were detected in a wide subtropical and temperate area of the country, while indigenous circulation of DENV occurred only in the subtropical area of the country. The first outbreak caused by DENV-2 occurred in northwestern Argentina in 1998; the second outbreak caused by DENV-1 occurred in the north-northeastern part of the country in 2000. Both outbreaks were preceded by DENV outbreaks in neighboring countries. The rates of DENV infections in neighboring countries varied widely. A serosurvey conducted in Bolivia in 1997 in a neighborhood of Santa Cruz City (10,000 inhabitants) showed that >5% of the populace was infected by DENV-2 (*5*). Paraguay’s Ministry of Health reported an infection rate of 49.3 cases per 10,000 inhabitants (including laboratory-diagnosed and reported cases) during a DENV-1 outbreak in 1999 and 2000 (6). Brazil reported incidence rates in different municipalities in 2000 that varied from 0.037 to 866.9 cases per 10,000 inhabitants (*9*). The geographic and climatologic situation in Argentina is favorable because most of the country has a temperate climate. Nevertheless, the introduction of different DENV serotypes increases the risk for dengue hemorrhagic fever because of the increased number of secondary DENV infections. The sequence of serotypes in secondary infections seems to be an important risk factor for dengue hemorrhagic fever because DENV-2 secondary infections account for most of those cases. Viral virulence related to specific genotypes is also thought to play an important role (*10*). All DENV cases that have occurred in Argentina to date have been considered clinically to be dengue fever. DENV-1 that circulated in Argentina during the period of study belongs to an American genotype closely related to viruses circulating previously in other American countries that have been associated only with mild disease (*8*). DENV-2 found in Argentina belongs to an Asian genotype that has been associated with severe disease (*11*). In Argentina, dengue hemorrhagic fever has not been reported, possibly because the size of the affected population was small and this complication occurs in a small proportion of secondary DENV infections (18–125/1,000) (*10*), and in an even smaller proportion of primary infections.

## References

[1] Boffi R. Programa de prevencion del dengue y control del *Aedes aegypti*. In: Temas de Zoonosis y enfermedades emergentes. 2do Congreso Argentino de Zoonosis, 1er Congreso Argentino y Latinoamericano de Enfermedades Emergentes y Asociacion Argentina de Zoonosis, editors (B. Aires); 1998. p. 413–9.

[2] Avilés G, Rangeon G, Vorndam V, Briones A, Baroni P, Enria D, Dengue reemergence in Argentina. Emerg Infect Dis. 1999;5:575–8. 10.3201/eid0504.990424 10460181 PMC2627740

[3] Avilés G, Rangeón G, Baroni P, Paz V, Monteros M, Sartini JL, Outbreak of dengue-2 virus in Salta, Argentina, 1998. Medicina (B Aires). 2000;60:875–9. 11436695

[4] Pan American Health Organization. Dengue and dengue hemorrhagic fever in the Americas: guidelines for prevention and control. PAHO scientific publication no. 548. Washington: the Organization; 1994. p. 1–21.

[5] Gianella A, Pirard M, Holzman A, Boelaert M, Fernandez-Ortiz F, Peredo C, Brote epidémico de denguevirus 2, genotipo Jamaica, en Bolivia. Salud Publica Mex. 1998;40:469–73. 10.1590/S0036-36341998000600002 9927881

[6] Boletín Epidemiológico. República del Paraguay. Ministerio de Salud Pública y Bienestar Social (Asunción). 2000;19:1–8.

[7] Avilés G, Rangeon G, Paz MV, Baroni P, Sabattini MS, Enria D. Secondary serologic responses to dengue virus during the 1998 outbreak in Salta, Argentina, where other flaviviruses co-circulate. Medicina (B Aires). 2001;61:129–36. 11374133

[8] Avilés G, Rowe J, Meissner J, Manzur Caffarena JC, Enria D, St. Jeor S. Phylogenetic relationships of dengue-1 viruses from Argentina and Paraguay. Arch Virol. 2002;147:2075–87. 10.1007/s00705-002-0886-3 12417945

[9] Barbosa da Silva J Jr, Siqueira JB Jr, Coelho GE, Vilarinhos PT, Pimenta FG Jr. Dengue in Brazil: current situation and prevention and control activities. Epidemiol Bull. 2002;23:3–6. 12197500

[10] Watts DM, Porter KR, Putvatana P, Vazquez B, Calampa C, Hayes CG, Failure of secondary infection with American genotype dengue 2 to cause dengue haemorrhagic fever. Lancet. 1999;354:1431–4. 10.1016/S0140-6736(99)04015-5 10543670

[11] Leitmeyer KC, Vaughn DW, Watts DM, Salas R, Villalobos de Chacon I, Ramos C, Dengue virus structural differences that correlate with pathogenesis. J Virol. 1999;73:4738–47. 10233934 10.1128/jvi.73.6.4738-4747.1999 PMC112516

